# Successful treatment for bilateral femoral neck insufficiency fractures: a rare lesion case report and an updated review of the literature

**DOI:** 10.1186/s12891-020-3107-x

**Published:** 2020-02-14

**Authors:** Xu-yi Tan, Ting Lei, Guan-bao Wu, Hai-en Luo, Gang Huang, Can-yu He, Min Lu, Peng-fei Lei

**Affiliations:** 1 Affiliated First Hospital of Hunan University of TCM, Changsha, 410007 Hunan China; 2 Affiliated Hospital of Hunan Academy of Chinese Medical Science, Changsha, 410006 Hunan China; 3 Xiangya Hospital of Centre-south University, Changsha, 410008 Hunan China

**Keywords:** Insufficiency fractures, Femoral neck fracture, Bilateral, Case report

## Abstract

**Background:**

The incidence of insufficiency fracture (IF) at femoral neck is low, accounting for about 5% of all insufficiency fractures, and IF at bilateral femoral neck is less common with more occurrence in athlete or serviceman. With the aging of populations, more cases of bilateral femoral neck IF have occurred recently, while the standard clinical treatment still remains lacking due to the complexity of these patients.

**Case presentation:**

A 55-year-old male patient complained pain in his bilateral hip, with no history of trauma, glucocorticoid hormone consumption or radiotherapy, and imaging examination revealed fracture nonunion and shortening in his left femoral neck, and double fracture line on the right femoral neck. The patient received a cementless THA for the left femoral neck fracture and conservative treatment for the right side, followed by Elcatonin injection and oral administration of Carbonate D3 Granules. After 4 months of fellow-up, the patient presented improved functional scorings in bilateral hip joints, with no signs of prothesis infection or loosening.

**Conclusion:**

We present a rare case of bilateral femoral neck IF in a middle-aged male and the treatment is successful. The timely CT and MRI examinations of bilateral hip joints for patients was necessary for orthopedists to select proper therapeutic regimen. In addition, the choice for therapeutic regimen of bilateral femoral IF should not only be based on the professional judgement of orthopedists, but also on the wishes of patients.

## Background

Insufficiency fractures (IF), which was first proposed by the professor Pentecost in 1964, was caused by normal or physiological stress applied to bone with decreased bone mineral content and deficient elastic resistance which would result in a weakened zone of the bone [1]. IF often occurs in the sacrum and ilium, while the incidence of IF at femoral neck is rare, accounting for about 5% of all stress fractures, not to mention bilateral femoral neck fracture [2–4]. Several femur neck IF cases have been reported [5], which generally occur in athlete and serviceman. Cases about femur neck IF occurring in other adult populations have been rarely reported, especially for bilateral femur neck IF. With the aging of populations, more cases of bilateral femoral neck IF have occurred recently, while the standard clinical treatment still remains lacking due to the complexity of these patients.

Herein, we presented a bilateral femur neck IF case of a middle-aged male, who suffered from bilateral femoral neck IF. Meanwhile, we also reported relevant treatment for the patient which could serve as a reference for other doctors.

## Case presentation

**Fig. 1:**
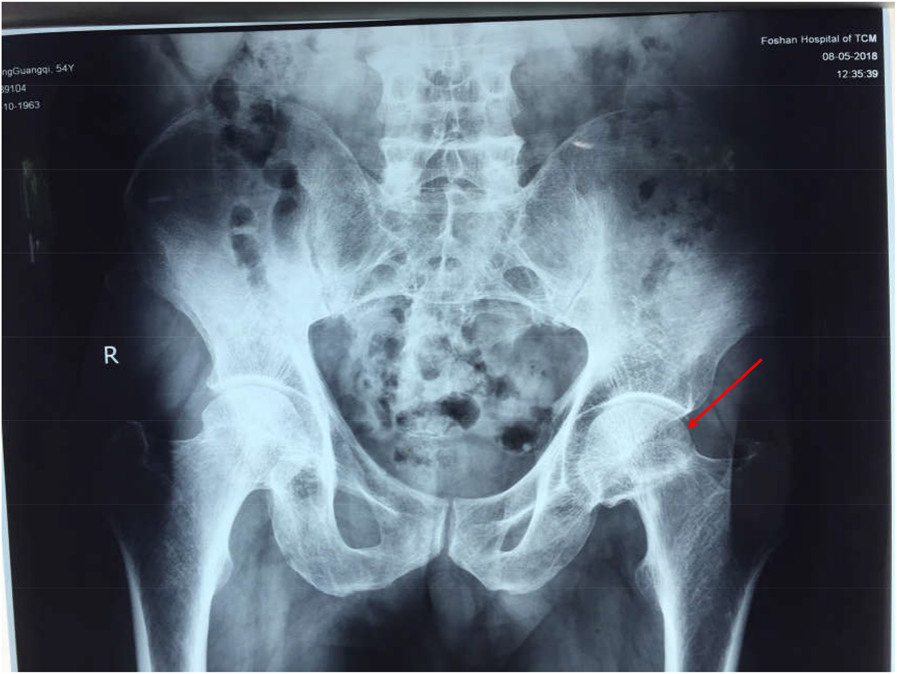
Plain radiograph of the pelvis indicating old fracture of the left femoral neck (red arrow) at a hospital in Foshan, Guangdong Province

**Fig. 2:**
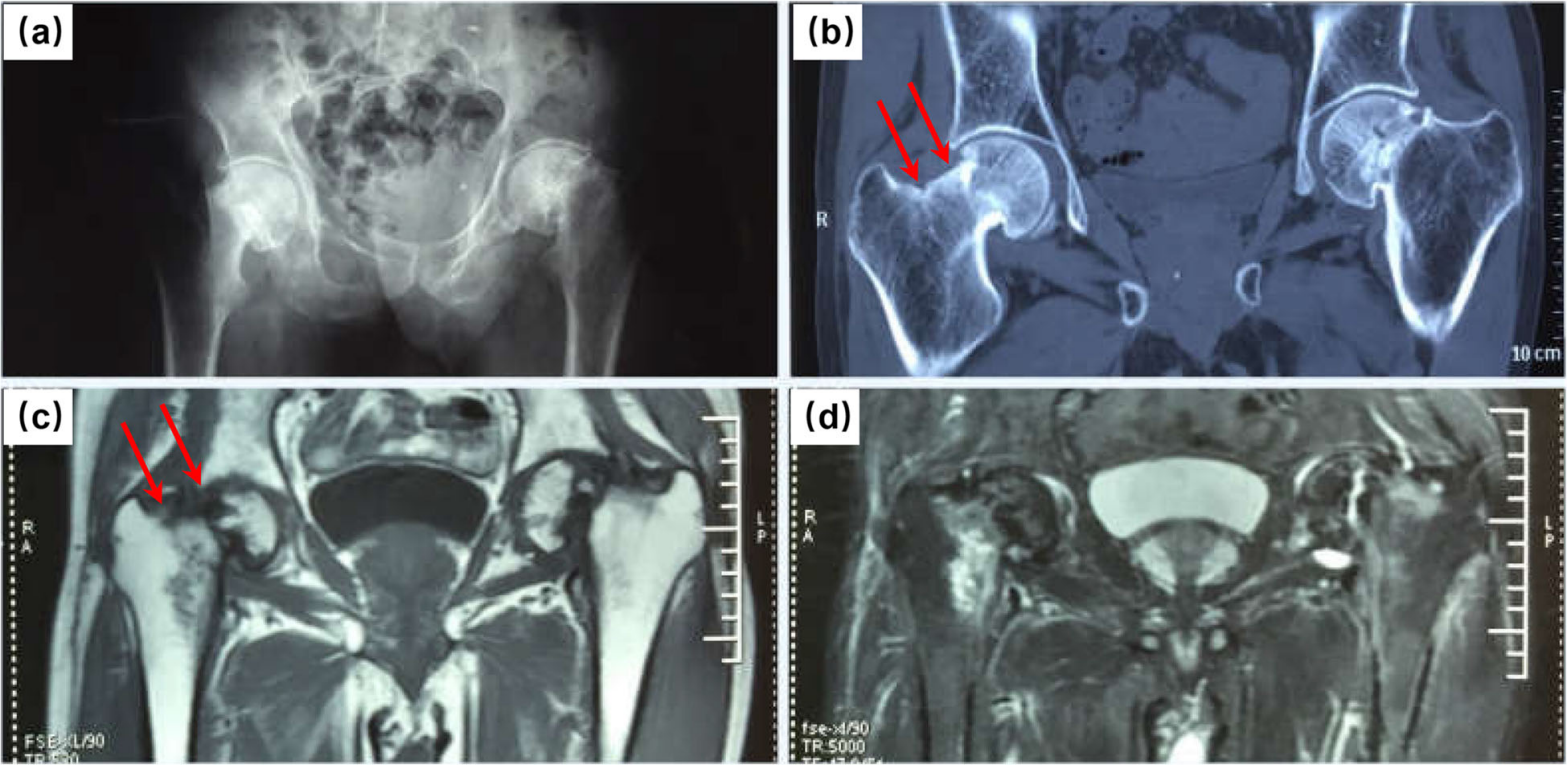
Imaging examinations of the patient in the hip region after admission into our hospital. **a** Plain radiograph of the pelvis indicating fracture of left femoral neck, with obvious displacement, nonunion and femoral neck shortening, and old fracture of the right femoral neck. **b** CT image of the coronal plane revealing bilateral femoral neck insufficiency fractures, with obvious displacement, nonunion and femoral neck shortening in the left femoral neck and double fracture line (arrows) in his right femoral neck. **c** Coronal T1-weighted image showing low signal intensity in the fracture region of bilateral femoral neck, with double fracture lines (arrows) on the right side. **d** Coronal T2-weighted image showing swelling on the right femoral neck and interruption of cortex on bilateral femoral neck

A 55-year-old man, with body mass index (BMI) of 18.36 kg/m2, was admitted into our hospital for left hip pain for 6 months. The symptom started with moderate pain after walking. Over the last few days, the pain got worsen with no remission after rest. The patient denied any history of trauma, glucocorticoid hormone consumption or radiotherapy. Examinations before admission revealed old fracture on the left femoral neck (Fig. 1) and an increase in blood uric acid. After admission, the function evaluations of his bilateral hip joint were summarized as follows: Visual Analogue Scale (VAS) score was 4, Harris score was 23 in the left hip joint, while VAS score was 0, Harris score was 90 in the right hip joint. The imaging examinations of the pelvis, including radiographs (Fig. 2a), CT (Fig. 2b) and MRI scan (Fig. 2c, d), confirmed the existence of bilateral femoral neck fractures, with fracture nonunion and femoral neck shortening in his left femoral neck and double fracture lines in his right femoral neck. The Dual energy X-ray absorptiometry (DXA) examination of the lumbar spine showed reduced bone mineral density (BMD) and severe osteoporosis (L1–L4: BMD0.514 g/cm2, T score = − 4.6). In addition, tumor markers, tuberculosis antibodies, alkaline phosphatase and parathyroid hormone levels were found to be normal.

**Fig. 3:**
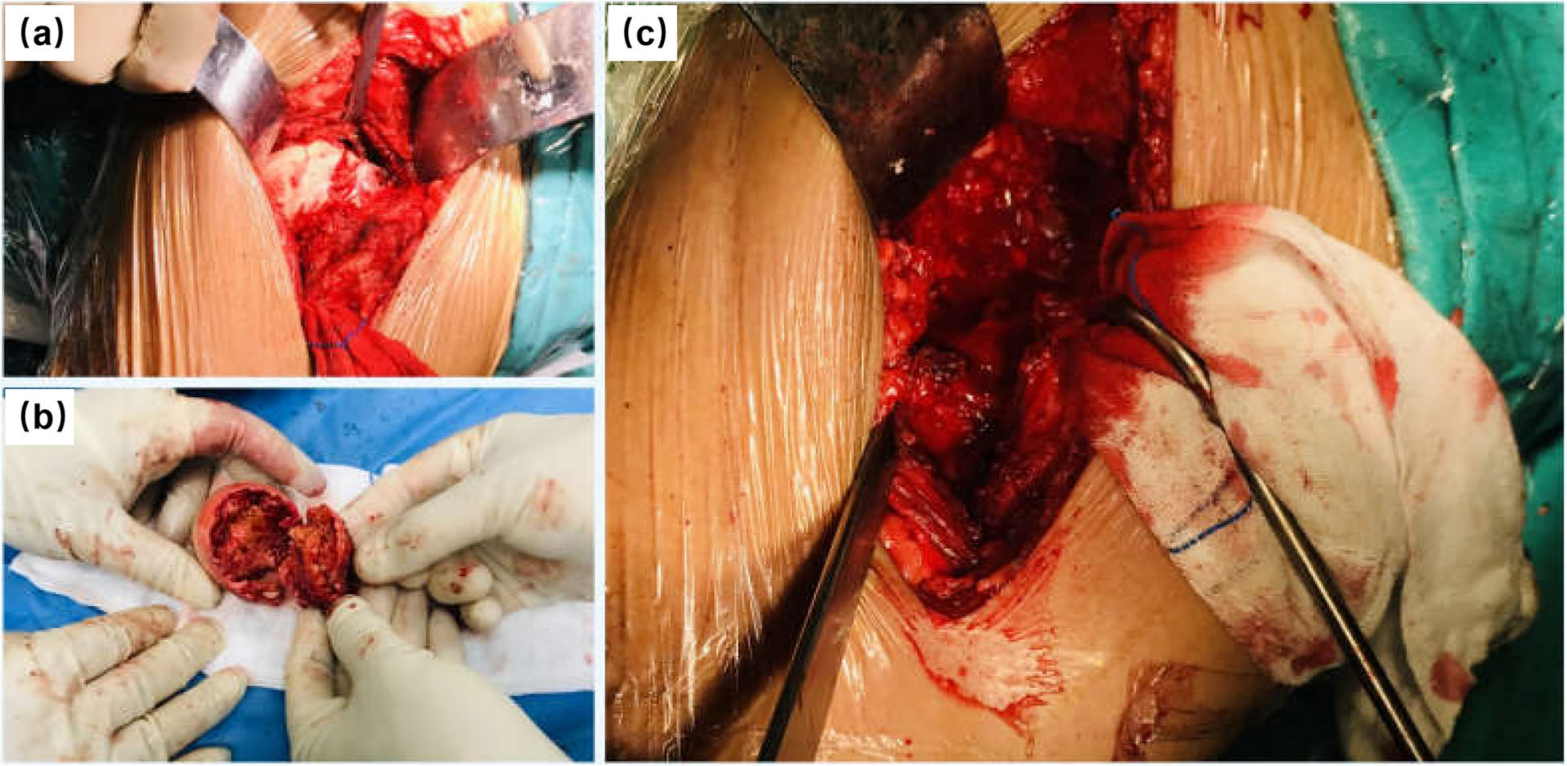
The operation procedure performed for the patient. **a** The lesion region revealed through the lateral approach of the left hip joint. **b** The necrosis bone tissue with fatty degeneration removed from the left femoral neck. **c** Impactive bone grafting was carry out due to acetabular bone mass was poor

We recommended a left cementless THA, and a cannulated screws fixation on the right side. And the uric acid lowering treatment was performed on the patient. However, the patient only agreed to the left THA, while on the right side, conservative treatment was selected for patient because of financial reasons. The left THA through the lateral approach was performed for the patient under general anesthesia (Fig. 3a). During the surgery, we found sclerosis, fatty degeneration and necrosis of the fracture end of the left femoral neck (Fig. 3b). We used impactive bone grafting to strengthen the acetabular on the fracture region (Fig. 3c).

**Fig. 4:**
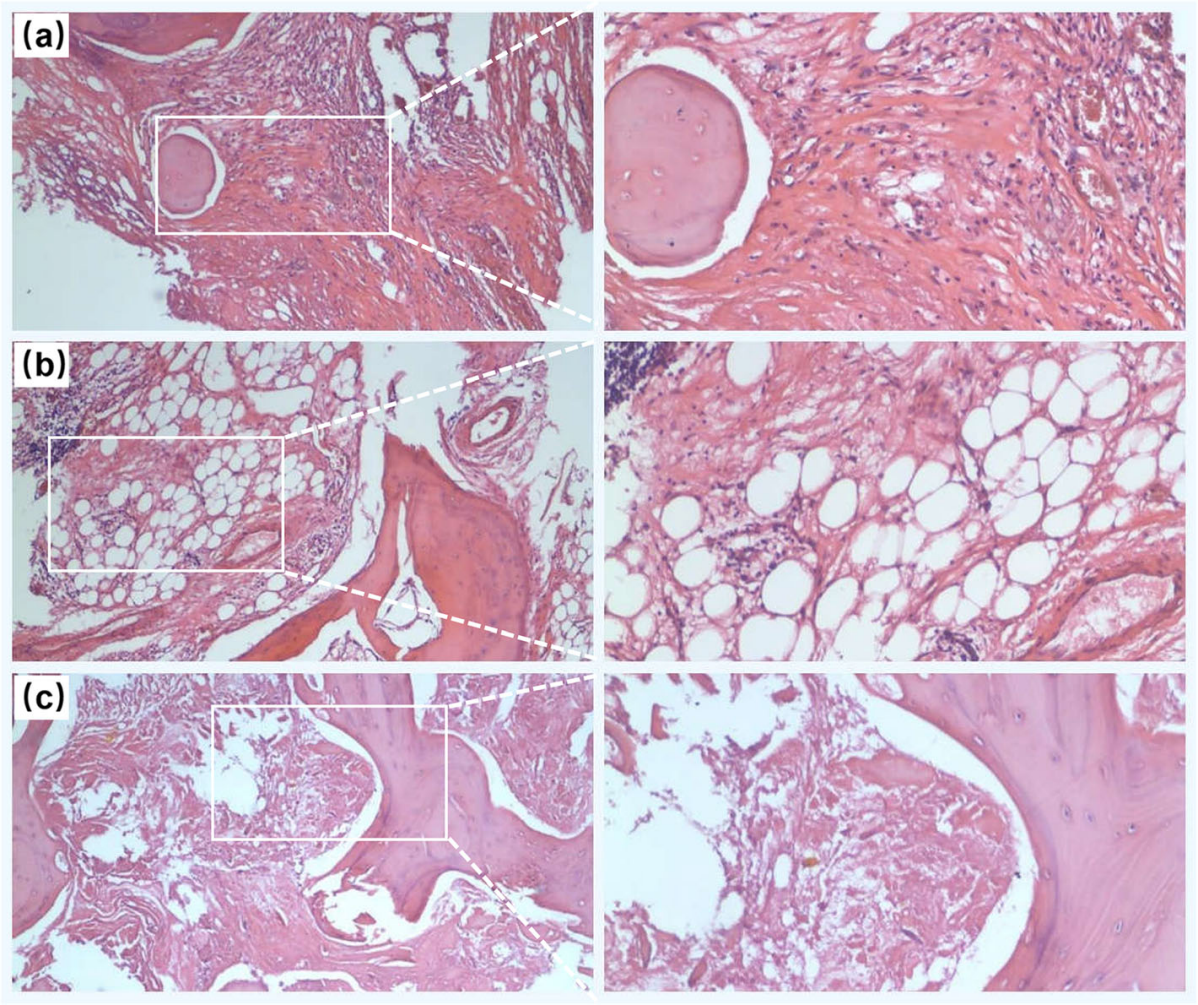
The HE staining pictures of bone tissue removed from the fracture region of the left femoral neck. **a** Some new but very few new bone tissues, with massive fibrous tissues, could be observed in the removed necrotic bone tissues. **b** some thinning trabeculae structure presented in some areas of the removed necrotic bone tissues. **c** Necrotic and fibrous bone tissues presented in most of the areas in the removed necrotic bone tissues. (magnification: 40 times)

**Fig. 5:**
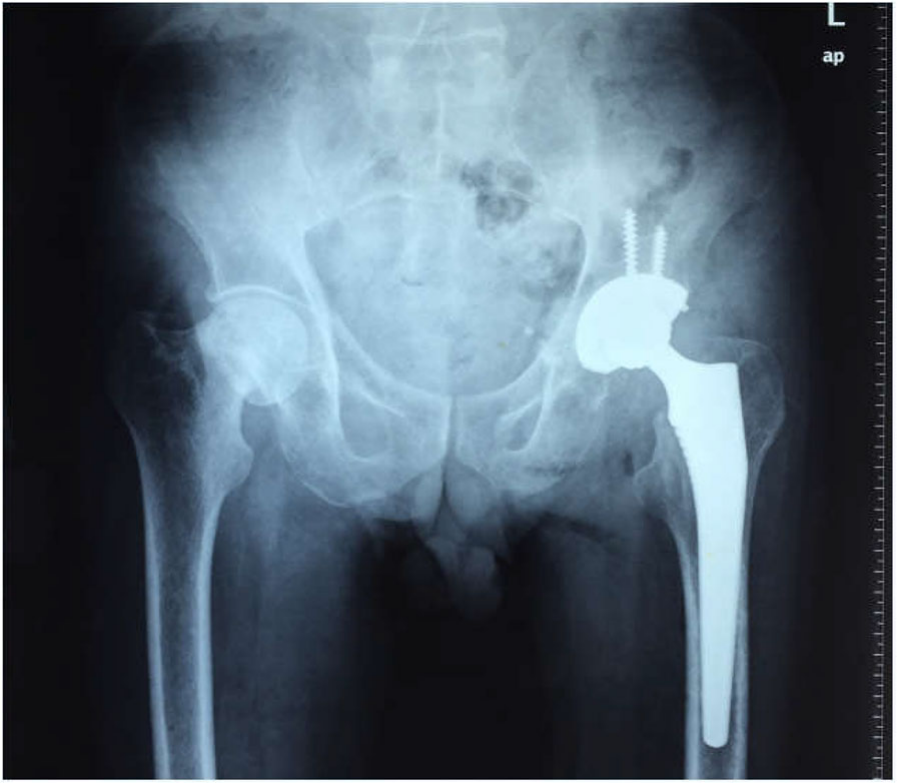
Postoperative radiograph of the pelvis demonstrating the success of the left total hip replacement

After the operation, the patient was treated with 20 U Elcatonin injection (Sidinuo®, Shangdong Luye Pharmaceutical Co.LTD, Yantai, China) once a week, and 1 tablet of Calcium Carbonate D3 Granules (Langdi®, Beijing Kangyuan Pharmaceutical CO.LTD, Beijing, China) containing calcium carbonate (500 mg) and vitamin D (200 IU), twice a day. Meanwhile, He was required to exercise his quadriceps and calf muscle on bed, move with the help of wheelchairs, and avoid walking on crutches. The postoperative histological examination, as showed in Fig. 4, revealed that the left fracture gap was filled with fibrous tissues, with few new bone tissues and a large amount of necrotic bone tissue observed. Meanwhile, the plain radiograph of the pelvis one day after surgery revealed that the total hip replacement arthroplasty was very successful (Fig. 5). Generally, the patient recovered well during the hospitalization and was successively discharged home.

**Fig. 6:**
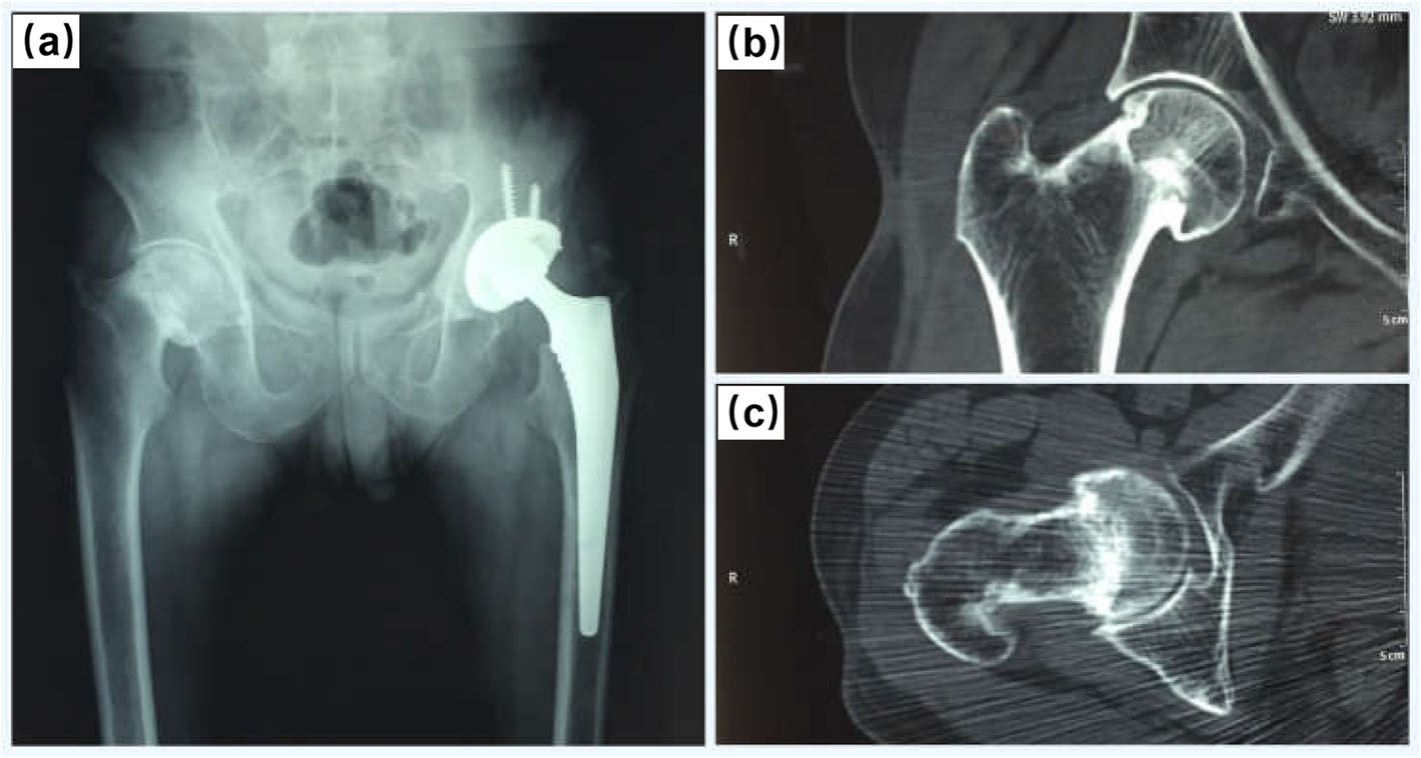
Imaging examination of the hip region for the patient four months after operation. **a** Radiograph of the pelvis showing well postoperative effect. **b, c** CT images of the right femoral neck showing fracture healing

Four months after discharge, the radiograph image showed no signs of implant loosening or infection in his left hip (Fig. 6a). It was worth noting that CT images revealed fracture union of the right femoral neck, as shown in Fig. 6b and Fig. 6c. In addition, function evaluations of his bilateral hips at four months after surgery was satisfactory and summarized as follows: VAS score was 0, Harris score was 98 in the left hip joint and VAS score was 0, Harris score was 95 in the right hip joint.

## Discussion and conclusion

Patients with IF often have multiple chronic diseases, such as osteoporosis, diabetes, chronic gastritis, chronic nephritis, organ transplantation, or rheumatoid arthritis needing immunosuppressive treatment, or tumor requiring radiation and chemotherapy. IF is more common in the elderly, and less common in men than women. Recently, Bakker et al [6] published a study about the diagnose of Sacral IF for 130 patients, who aged between 46 and 98 years (mean, 79.8 years), with 117 females and 13 males. Melton et al [7] analyzed the contribution of gender on the incidence of pelvic insufficiency fractures in a retrospective review of a Mayo Medical database, of which total of 198 patients with 204 fractures, showed the incidence of pelvic insufficiency fractures was nearly twice as common in women (47.5/100000) as it is in men (24.4/100000).

IF was produced by normal or physiological stress applied to bone with decreased bone mineral content and deficient elastic resistance, resulting in a weakened zone of the bone. As such, this kind of fracture are often caused by daily activities or even light exercise, and usually are not associated with trauma [8]. Osteoporosis is the most common predisposing factor for IF [9]. Bisphosphonates are considered the first-line therapy of postmenopausal osteoporosis, as they could improve bone density and inhibit bone resorption. However, several reports have suggested an association between the use of bisphosphonates and subtrochanteric IF in recent years, and recommended that care should be taken when using bisphosphonates for more than 5 years [10–12].

Due to the special characteristics and atypical basic clinical manifestations of IF, it is difficult to be diagnosed by conventional imaging examination. Therefore, IF is often misdiagnosed, resulting in delay of treatment. The findings from conventional radiographs often appear to be normal early in the course of IF. However, CT could depict subtle fracture lines allowing direct visualization of cortical and trabecular bone. MRI is a very sensitive tool to visualize bone marrow abnormalities associated with insufficiency fractures, and could help distinguish between benign and malignant fractures [13].

IF often occur in the sacrum and ilium. Therefore, IF at femoral neck are unusual, accounting for about 5% of all stress fractures. Bilateral femoral neck fractures are even more rare [2–4]. We find several cases about bilateral femoral neck fractures. Kalaci et al [14] described a case of a 18-year-old girl with bilateral femoral neck IF. The girl was treated surgically with in-situ internal fixation using cannulated screws. Baki ME et al [15] reported a 22-year-old female case with bilateral femoral neck fractures, of whom the diagnosis was delayed because the patient was pregnant and could not receive imaging examination. Finally, the female patient was treated surgically with internal fixation using cannulated screws and received medical treatment for vitamin D deficiency. Ahn DK et al [16] reported a bilateral femoral neck IF case of a 78-year-old woman who had a long history of using anti-resorptive drug, and bilateral internal fixations using cannulated screws were performed for the patient. Vaishya R et al [2] reported a 50-year old male patient who simultaneously suffered from chronic kidney disease and bilateral femoral neck IF with minor trochanter IF. Finally, the patient was managed with cannulated screws at unusual sites.

In our case, the patient had a 7-year history of drinking, about 60 g per day, and had a poor appetite with few breakfasts in daily life. These bad life styles possibly caused malnutrition and finally contributed to the development of bilateral femoral neck IF [17]. Although no examinations related to nutrition evaluations were carried out, such as Vitamin D, the reduced BMD of his lumbar spine reflected a poor nutrition condition of the patient, which was a potential risk for the occurrence of femoral neck IF. However, the poor nutrition condition cannot fully explain the occurrence of bilateral femoral fracture. We suspected that the patient had fallen or done physically demanding work, while the patient denied this which he may forget.

In conclusion, from our experience with the patient with bilateral femoral neck IF, he complained of pain in left hip when walking, which led to suspicion of a femoral neck fracture. Although radiological examination was performed and indicated old fracture of the left femoral neck. The immediate CT and MRI examination was not followed for further examination, which is considered great value to evaluate fracture healing. Because of the pain on the left side, the upper body weight overload on the right side may have caused the subsequent IF on the right side. Due to the increase of the average age of the population, as the incidence of IF is increasing, it is of great importance to perform CT and MRI scan additionally.

## Data Availability

All relevant data was presented within the manuscript and the datasets used and/or analyzed during the current study are available from the corresponding author on reasonable request.

## Abbreviations

BMD: Bone mineral density
CT: Computed tomography
IF: Insufficiency Fracture
MRI: Magnetic resonance imaging
THA: Total hip arthroplasty
